# Supplementary material for: De novo genome assembly resolving repetitive structures enables genomic analysis of 35 European Mycoplasmopsis bovis strains
Source: BMC Genomics. 2023 Sep 16;24:548. doi: 10.1186/s12864-023-09618-5 (PMC10504702; doi:10.1186/s12864-023-09618-5)
Supplement: Supplementary file 1 — Additional file 1. [file 12864_2023_9618_MOESM1_ESM.pdf]

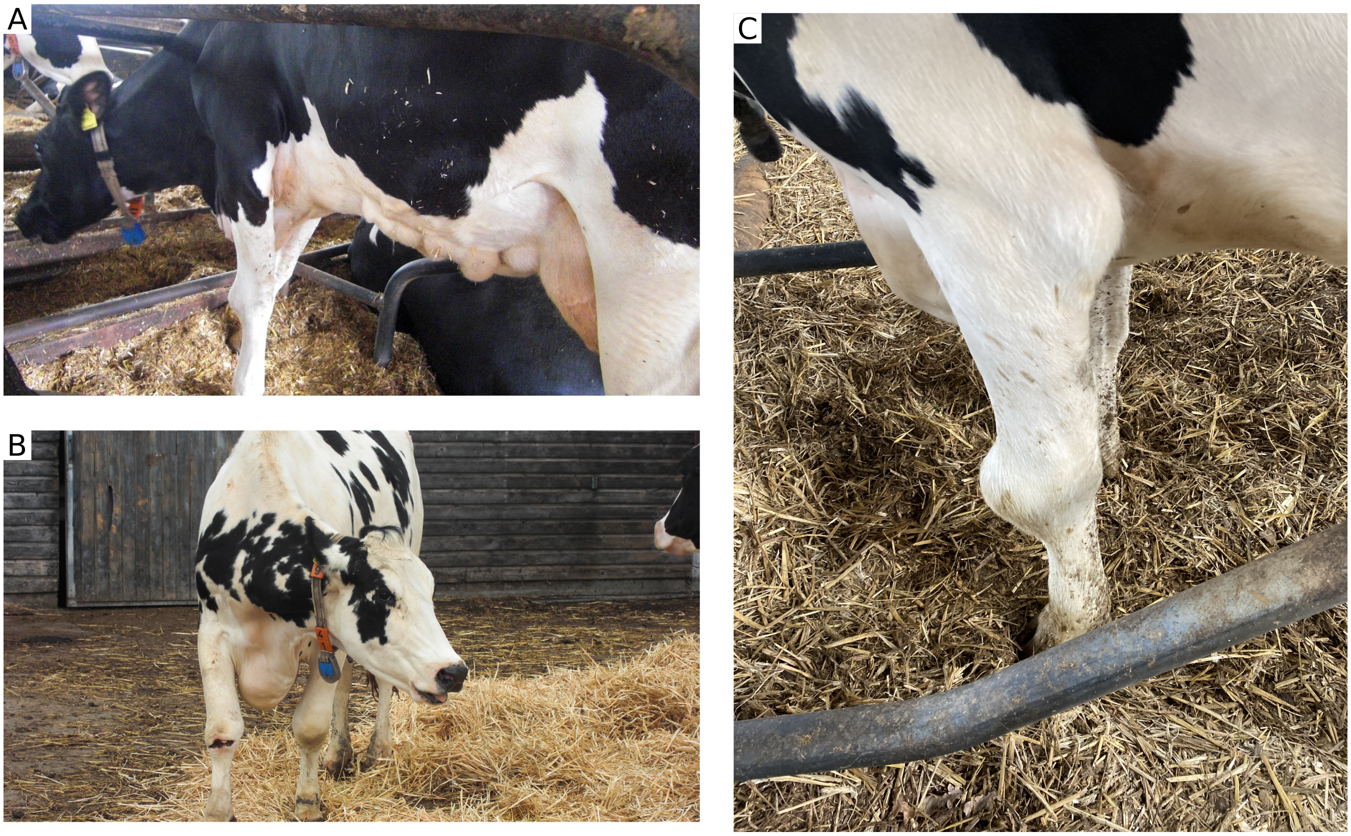

Figure S1. *M. bovis* infection causing systemic circular condition (SCC) showing signs of edema and arthritis. (A) Edema in the abdominal area, (B) Severe edema in the chest area, (C) Arthritis of the front leg.

Table S1. General assembly statistics of 36 *M. bovis* strains. Bio Information – Biological Information; Ref – RefSeq; DC – Diary Cow; AC – Adult cattle; Ca – Calf; Co – Cow; DNA concentration in ng/µl (Nanodrop and Qubit); #Reads – number of raw reads; MeanL – mean length of raw reads; F – flow cell number; LR – length of longest read (and its quality) in bp; P – processed; #C – number of contigs after assembly; Total L – length of total assembly in bp; L Contg – length of longest contig in bp; GC – GC content (%); CDS – number of annotated CDS (including unknown proteins); CDS* – number of CDS with annotated gene name (without hypothetical proteins and putative product names); tR – number of tRNAs; rR – number of rRNAs; tm – number of tmRNAs; Core – number of core genes; SCore – number of soft core genes (present in at least 0.95 % of the genomes) Shell – number of shell genes (presence thresholds - min: 0.22, max: 0.81, sd: 0.17, mean: 0.55); Cloud – number of cloud genes (presence thresholds - min: 0.03, max: 0.53, sd: 0.09, mean: 0.08);

Bio Information				DNA concentration		Illumina Information		ONT Information					General Assembly Statistics				Annotation Statistics					Pangenome Statistics			
		Year	Clade	Nanodrop	Qubit	#Reads	MeanL	F	#Reads	MeanL	N50	LR (quality)	#C	Total L	L Contg	GC	CDS	CDS*	tR	rR	tm	Core	SCore	Shell	Cloud
Ref 8790													17	905,797	254,357	29.50	742		34	4	1				
Ref PG45			6.2										1	1,003,404	1,003,404	29.30	829		34	6					
DL422_88	Ca	1988	0	172.20	16.70	22,148,828	126	4	249,877	7,129.1	16,243	133,702 (14.2)	1	1,046,374	1,046,374	29.20	871	355	34	6	1	598	629	28	84
15DD0210		2015	6.2	97.00	9.00	11,356,887	126	3	491,868	2,429.0	4,334	109,119 (9.4)	1	1,017,740	1,017,740	29.29	853	364	34	6	1	598	632	17	46
15DD0261	Ca	2015	6.1	322.25	32	9,552,559	151	3	364,896	1,884.8	4,170	102,976 (9.2)	1	1,026,147	1,026,147	29.30	856	354	34	6	1	598	630	23	95
15DD0240	Ca	2014		163.50	6.90	7,709,202	126	4	204,408	3,728.3	14,068	227,226 (11.1)	1	953,284	953,284	29.37	797	354	36	6	1	598	628	13	50
15DD0263	C	2015		134.40	8.60	12,082,542	126	4	180,976	4,174.2	12,812	180,080 (10.5)	3	1,046,052	908,152	29.39	888	371	38	6	1	598	631	24	56
17DD0020	Co	2017	6.3	319.79	7.52	6,893,910	151	1	229,537	803.0	985	41,015 (11.5)	16	990,775	330,523	29.25	855	356	34	4	1	598	632	8	59
DL81_99		1999		393.61	10.10	8,215,831	151	4	240,108	2,722.5	5,861	122,336 (9.3)	1	1,044,487	1,044,487	29.41	888	352	34	6	1	598	634	23	93
17DD0007		2017		319.79	7.52	7,165,832	151	1	369,101	732.9	908	42,771 (11.8)	1	991,212	991,212	29.27	837	351	34	4	1	598	633	13	55
16DD0054	Ca	2016		252.10	7.70	1,716,256	126	4	241,014	3,434.5	5,438	132,662 (9.8)	1	996,378	996,378	29.43	825	345	34	6	1	598	630	29	28
15DD0250	Ca	2014		162.40	12.50	13,235,007	126	4	125,566	4,476.8	14,729	170,021 (13.8)	1	1,027,924	1,027,924	29.35	860	354	34	6	1	598	634	42	26
15DD0163	Ca	2015		238.90	36.50	8,316,899	126	3	98,914	5,206.0	12,346	125,285 (14.5)	2	1,045,717	1,040,623	29.36	870	357	35	6	1	598	634	41	24
15DD0207		2015		248.10	12.20	10,222,109	126	3	252,526	4,304.4	8,400	121,699 (9.2)	1	1,053,933	1,053,933	29.36	875	361	35	6	1	598	634	39	23
16DD0100		2016		271.10	10.10	8,085,183	126	4	116,974	6,963.2	18,046	210,665 (11.1)	1	1,002,935	1,002,935	29.34	835	351	34	6	1	598	634	38	21
15DD0123		2015	5	132.10	11.00	8,815,543	126	2	257,173	4,534.8	10,474	170,872 (10.4)	1	1,010,945	1,010,945	29.35	841	351	34	6	1	598	634	37	28
15DD0140		2015		120.70	14.50	7,206,604	126	2	500,382	3,080.2	6,389	152,850 (9.6)	2	1,015,460	986,739	29.41	850	350	34	6	1	598	633	38	33
15DD0233		2015		271.60	10.60	5,987,289	126	3	177,766	3,550.0	7,443	100,036 (10.0)	1	1,005,274	1,005,274	29.36	840	348	34	6	1	598	634	40	24
16DD0001		2016		445.22	16.4	5,497,038	151	4	172,515	4,334.7	12,277	147,403 (9.3)	1	1,011,582	1,011,582	29.37	843	351	34	6	1	598	634	39	30
15DD0228		2015		342.20	9.28	7,896,760	126	3	596,225	1,696.5	3,687	161,386 (3.7)	1	971,138	971,138	29.34	804	349	34	6	1	598	634	27	22
15DD0165		2015		213.00	7.84	7,013,238	126	3	323,361	2,961.5	5,992	133,582 (13.0)	1	1,010,634	1,010,634	29.39	848	351	34	6	1	598	634	42	30
15DD0249	Ca	2014		147.60	9.78	7,483,705	126	4	179,761	6,841.2	18,142	188,317 (10.2)	1	1,065,918	1,065,918	29.22	890	352	34	6	1	598	632	89	4
11DD0261	Co	2011		196.90	4.39	9,498,037	126	2	263,161	4,678.6	8,786	140,830 (8.6)	1	1,122,166	1,122,166	29.15	932	360	34	6	1	598	634	93	7
14DD0148	Bu	2014		120.60	10.90	10,052,897	126	2	60,465	2,836.0	6,760	120,138 (14.1)	2	1,095,392	1,090,223	29.22	907	353	35	6	1	598	633	89	11
15DD0161	Co	2015		69.00	7.86	7,910,036	126	2	472,501	3,305.6	8,117	320,318 (5.5)	1	1,091,493	1,091,493	29.19	912	352	34	6	1	598	633	80	8
14DD0475		2014		329.26	15.4	7,181,419	151	2	525,440	2,133.0	3,920	173,043 (12.2)	1	1,077,003	1,077,003	29.28	896	353	34	8	1	598	634	93	8
14DD0156	Ca	2014		199.10	14.50	10,609,103	126	2	909,463	3,103.3	5,784	461,771 (7.2)	4	1,073,809	576,443	29.19	889	354	34	6	1	598	633	88	9
15DD0238	DCo	2015		136.60	8.10	11,278,154	126	3	608,083	2,098.8	3,761	127,525 (14.3)	1	1,091,568	1,091,568	29.19	908	352	34	6	1	598	633	87	10
15DD0234	Co	2015		106.90	6.70	10,832,930	126	3	215,402	3,602.3	8,541	119,027 (10.3)	1	1,105,287	1,105,287	29.13	919	352	34	6	1	598	633	82	11
15DD0218		2018	4	59.00	7.36	10,443,706	126	3	419,865	1,672.5	2,671	124,420 (9.8)	1	1,135,727	1,135,727	29.16	946	352	34	8	1	598	634	84	17
15DD0164		2015		199.20	17.00	9,059,621	126	3	952,993	1,966.5	3,699	116,403 (12.1)	1	1,133,513	1,133,513	29.15	952	352	34	6	1	598	634	85	12
15DD0141		2015		183.40	14.20	8,812,480	126	2	427,464	3,600.9	7,303	170,001 (14.7)	1	1,068,782	1,068,782	29.21	899	353	34	6	1	598	633	94	7
15DD0160	Ca	2015		154.50	13.30	10,174,035	126	2	349,154	3,713.5	7,441	156,846 (13.4)	1	1,098,927	1,098,927	29.20	920	353	34	6	1	598	634	82	8
16DL0615		2016		402.87	14.60	8,347,668	151	4	233,156	3,437.4	9,291	124,555 (12.2)	1	1,141,547	1,141,547	29.13	962	358	34	6	1	598	634	95	9
14DD0147	Co	2014		164.50	31.00	10,294,081	126	3	307,197	5,333.0	13,275	315,817 (12.4)	1	1,071,008	1,071,008	29.22	892	352	34	6	1	598	634	92	7
16DD0186		2016		70.90	8.54	13,582,089	126	4	193,932	6,233.6	14,319	178,119 (12.2)	1	1,089,251	1,089,251	29.19	912	352	34	6	1	598	634	92	11
15DL0124		2015		575.33	13.40	10,749,259	151	4	94,911	6,966.1	16,334	169,771 (9.5)	1	1,081,338	1,081,338	29.19	906	352	34	6	1	598	634	92	13
13DD0918	Co	2013		84.50	6.80	11,512,328	126	2	38,551	4,496.2	11,416	134,390 (9.7)	1	1,084,984	1,084,984	29.24	907	353	34	6	1	598	634	91	15

Table S2. Virulence and *vsp* genes. The presence of virulence genes is based on the `Prokka` annotation without reference where the *vsp* gene count is derived from the reference-based annotations.

Bio Information	Virulence Gene Information																									
	infC	tuf	fusA	rpsL	rpsG	rpmI	rplT	rpoB	rpoC	gyrA	gyrB	ulaB	ulaC	oppD	uvrC	pta	secA	tkt	xerC	eno2	ldh1	gls(C)	malF	pyk	ndh	clpC
Ref 8790	1	1	1	1	1	1	1	0	1	1	0	0	0	0	0	2	1	0	0	0	1	0	0	1	0	0
Ref PG45	1	1	1	1	1	1	1	1	1	1	1	0	0	1	1	2	1	1	0	0	1	0	0	1	0	0
DL422_88	1	1	1	1	1	1	1	1	1	1	1	1	1	1	1	2	1	1	2	1	1	0	0	0	0	0
15DD0210	1	1	1	1	1	1	1	1	1	1	1	1	1	1	2	2	1	1	3	1	1	0	0	0	0	0
15DD0261	1	1	1	1	1	1	1	1	1	1	1	1	1	1	1	2	1	1	4	1	1	0	0	0	0	0
15DD0240	1	1	1	1	1	1	1	1	1	1	1	1	1	1	1	2	1	1	3	1	1	0	0	0	0	0
15DD0263	1	1	1	1	1	1	1	1	1	1	2	1	1	1	1	2	1	1	4	1	1	0	0	0	0	0
17DD0020	2	1	1	1	1	1	1	1	1	1	1	1	1	1	1	2	1	1	3	1	1	0	0	0	0	0
DL81_99	1	1	1	1	1	1	1	1	1	1	1	1	1	1	1	2	1	1	4	1	1	0	0	0	0	0
17DD0007	1	1	1	1	1	1	1	1	1	1	1	1	1	1	1	2	1	1	3	1	1	0	0	0	0	0
16DD0054	1	1	1	1	1	1	1	1	1	1	1	1	1	1	1	2	1	1	3	1	1	0	0	0	0	0
15DD0250	1	1	1	1	1	1	1	1	1	1	1	1	1	1	1	2	1	1	3	1	1	0	0	0	0	0
15DD0163	1	1	1	1	1	1	1	1	1	1	1	1	1	1	1	2	1	1	4	1	1	0	0	0	0	0
15DD0207	1	1	1	1	1	1	1	1	1	1	1	1	1	1	1	2	1	1	4	1	1	0	0	0	0	0
16DD0100	1	1	1	1	2	1	1	1	1	1	1	1	1	1	1	2	1	1	3	1	1	0	0	0	0	0
15DD0123	1	1	1	1	1	1	1	1	1	1	1	1	1	1	1	2	1	1	3	1	1	0	0	0	0	0
15DD0140	1	1	1	1	1	1	1	1	1	1	1	1	1	1	1	2	1	1	3	1	1	0	0	0	0	0
15DD0233	1	1	1	1	1	1	1	1	1	1	1	1	1	1	1	2	1	1	3	1	1	0	0	0	0	0
16DD0001	1	1	1	1	1	1	1	1	1	1	1	1	1	1	1	2	1	1	3	1	1	0	0	0	0	0
15DD0228	1	1	1	1	1	1	1	1	1	1	1	1	1	1	1	2	1	1	3	1	1	0	0	0	0	0
15DD0165	1	1	1	1	1	1	1	1	1	1	1	1	1	1	1	2	1	1	3	1	1	0	0	0	0	0
15DD0249	1	1	1	1	1	1	1	1	1	1	1	1	1	1	1	2	1	1	2	1	1	0	0	0	0	0
11DD0261	1	1	1	1	1	1	1	3	2	1	1	1	1	1	1	2	1	1	2	1	1	0	0	0	0	0
14DD0148	1	1	1	1	1	1	1	1	1	1	1	1	1	1	1	2	1	1	2	1	0	0	0	0	0	0
15DD0161	1	1	1	1	1	1	1	1	1	1	1	1	1	1	1	2	1	1	2	1	1	0	0	0	0	0
14DD0475	1	1	1	1	1	1	1	1	1	1	1	1	1	1	1	2	1	1	2	1	1	0	0	0	0	0
14DD0156	1	1	1	1	1	1	1	1	1	1	1	1	1	1	1	2	1	1	2	1	1	0	0	0	0	0
15DD0238	1	1	1	1	1	1	1	1	1	1	1	1	1	1	1	2	1	1	2	1	1	0	0	0	0	0
15DD0234	1	1	1	1	1	1	1	1	1	1	1	1	1	1	1	2	1	1	2	1	1	0	0	0	0	0
15DD0218	1	1	1	1	1	1	1	1	1	1	1	1	1	1	1	2	1	1	2	1	1	0	0	0	0	0
15DD0164	1	1	1	1	1	1	1	1	1	1	1	1	1	1	1	2	1	1	2	1	1	0	0	0	0	0
15DD0141	1	1	1	1	1	1	1	1	1	1	1	1	1	1	1	2	1	1	2	1	1	0	0	0	0	0
15DD0160	1	1	1	1	1	1	1	1	1	1	1	1	1	1	1	2	1	1	2	1	1	0	0	0	0	0
16DL0615	1	1	1	1	1	1	1	1	1	1	1	1	1	1	1	2	1	1	2	1	1	0	0	0	0	0
14DD0147	1	1	1	1	1	1	1	1	1	1	1	1	1	1	1	2	1	1	2	1	1	0	0	0	0	0
16DD0186	1	1	1	1	1	1	1	1	1	1	1	1	1	1	1	2	1	1	2	1	1	0	0	0	0	0
15DL0124	1	1	1	1	1	1	1	1	1	1	1	1	1	1	1	2	1	1	2	1	1	0	0	0	0	0
13DD0918	1	1	1	1	1	1	1	1	1	1	1	1	1	1	1	2	1	1	2	1	1	0	0	0	0	0

Bio Information	*vsp* 8790-based Annotation																	
	WP_233435476	WP_211235702	WP_136832249	WP_233435477	WP_136832641	WP_136832072	WP_136832633	WP_136831985	WP_136832637	WP_136832228	WP_136831481	WP_136831846	WP_233435459	WP_136831431	WP_136831844	WP_136832078	WP_13683208	WP_136832058
Ref 8790	1	1	1	1	1	1	1	1	1	1	1	1	1	1	1	1	1	1
Ref PG45	0	0	0	0	0	0	0	0	0	0	0	0	0	0	0	0	0	0
DL422_88	1	0	0	0	0	0	0	0	0	1	0	2	1	2	2	1	1	2
15DD0210	1	0	0	0	0	0	0	1	0	1	0	0	0	1	1	1	1	2
15DD0261	1	1	0	0	0	0	0	1	1	1	0	0	0	1	1	1	1	2
15DD0240	1	0	0	0	0	0	1	1	0	1	0	0	0	1	2	1	1	2
15DD0263	2	0	0	2	0	0	0	1	0	1	0	0	0	1	1	1	1	2
17DD0020	1	0	0	1	0	0	0	0	0	1	0	0	0	1	1	1	1	2
DL81_99	1	1	0	0	0	0	0	1	0	1	0	0	0	1	1	1	1	3
17DD0007	1	0	0	0	0	0	0	1	0	1	0	0	0	1	1	1	1	2
16DD0054	1	0	0	0	0	0	0	0	0	0	0	0	0	2	1	1	1	2
15DD0250	1	0	0	0	0	0	0	0	0	0	0	1	0	2	1	1	1	2
15DD0163	2	0	0	1	1	0	0	0	0	0	0	1	0	2	1	1	1	2
15DD0207	2	0	0	0	0	0	0	0	0	0	0	1	0	2	1	1	1	2
16DD0100	1	0	0	0	0	0	0	0	0	0	0	1	0	2	1	1	1	2
15DD0123	1	0	0	0	0	0	0	0	0	0	0	1	0	2	1	1	1	2
15DD0140	1	0	0	2	0	0	0	0	0	0	0	1	0	2	1	1	1	3
15DD0233	1	0	0	0	0	0	0	0	0	0	0	1	0	2	1	1	1	2
16DD0001	1	0	0	1	0	0	0	0	0	0	0	1	0	2	1	2	1	2
15DD0228	1	0	0	0	0	0	0	0	0	0	0	1	0	2	1	1	1	2
15DD0165	1	0	0	0	0	0	0	0	0	0	0	1	0	2	1	1	1	2
15DD0249	1	0	0	0	0	0	0	1	1	0	0	0	0	2	0	1	1	2
11DD0261	1	0	0	0	0	0	0	1	1	0	0	1	0	2	1	1	1	2
14DD0148	1	0	0	0	0	0	1	1	1	0	0	1	0	2	1	1	1	2
15DD0161	1	0	0	0	0	0	1	0	1	0	0	1	0	2	1	1	1	2
14DD0475	1	0	0	0	0	0	0	1	1	0	0	1	0	2	1	1	1	2
14DD0156	1	0	0	0	0	0	0	1	1	0	0	1	0	2	1	1	1	2
15DD0238	1	0	0	0	0	0	0	1	1	0	0	1	0	2	1	1	1	2
15DD0234	1	0	0	0	0	0	0	1	1	0	0	2	0	2	1	1	1	2
15DD0218	1	0	0	0	0	0	0	0	1	0	0	1	0	2	1	1	1	2
15DD0164	1	0	0	0	0	0	0	0	2	0	0	1	0	2	1	1	1	2
15DD0141	1	0	0	0	0	0	0	1	0	0	0	1	0	2	1	1	1	2
15DD0160	1	0	0	0	0	0	0	0	1	0	0	0	0	2	1	1	1	2
16DL0615	1	0	0	0	0	0	0	1	1	0	0	1	0	2	2	2	1	2
14DD0147	0	0	0	0	0	0	0	1	1	0	0	1	0	2	1	1	1	2
16DD0186	1	0	0	0	0	0	0	1	1	0	0	1	0	2	1	1	1	2
15DL0124	1	0	0	0	0	0	0	1	1	0	0	1	0	2	1	1	1	2
13DD0918	1	0	0	0	0	0	0	1	1	0	0	1	0	2	1	1	1	2

Bio Information	*vsp* PG45-based Annotation												
	A	B	E	F	G	H	I	J	K	L	M	N	O
Ref 8790	0	0	0	0	0	0	0	0	0	0	0	0	0
Ref PG45	1	1	1	1	1	1	1	1	1	1	1	1	1
DL422_88	1	0	0	0	1	0	0	0	0	1	0	0	0
15DD0210	2	2	0	0	1	1	0	0	1	0	0	0	0
15DD0261	0	0	0	0	2	0	0	1	1	1	0	0	1
15DD0240	1	0	0	0	1	1	1	0	2	0	0	1	0
15DD0263	9	0	0	0	1	0	0	0	1	3	0	0	0
17DD0020	4	0	0	0	0	0	0	0	0	1	0	0	0
DL81_99	7	0	0	0	0	0	0	0	1	1	0	0	0
17DD0007	5	0	0	0	1	1	0	0	1	1	0	0	0
16DD0054	0	0	0	0	2	0	2	1	0	0	0	2	2
15DD0250	0	0	0	0	2	1	1	1	1	1	0	2	1
15DD0163	1	0	0	0	1	4	0	1	3	0	0	4	2
15DD0207	0	0	0	0	0	4	0	1	4	0	0	4	3
16DD0100	0	0	0	0	1	1	0	1	1	1	0	3	2
15DD0123	0	0	0	0	2	1	1	1	1	1	0	2	2
15DD0140	0	0	0	0	2	1	1	2	1	1	0	2	3
15DD0233	0	0	0	0	2	1	1	1	1	1	0	2	2
16DD0001	0	0	0	0	2	2	1	1	1	1	0	2	3
15DD0228	1	0	0	0	1	0	0	1	0	1	0	1	0
15DD0165	0	0	0	0	2	1	1	1	1	1	0	2	3
15DD0249	1	0	1	0	2	0	1	0	2	1	1	1	0
11DD0261	1	0	1	0	2	0	1	0	1	1	1	1	0
14DD0148	3	0	2	0	2	0	1	0	1	1	1	4	0
15DD0161	1	0	1	0	1	1	1	0	1	1	1	1	0
14DD0475	1	0	1	0	3	1	1	0	1	1	1	1	0
14DD0156	0	0	0	0	1	0	1	0	1	1	0	1	0
15DD0238	1	0	1	0	1	0	1	0	1	1	1	1	1
15DD0234	1	0	1	0	2	0	1	0	1	1	1	0	0
15DD0218	2	0	0	0	3	0	0	0	2	2	1	2	0
15DD0164	1	0	0	0	3	0	0	0	3	2	2	3	0
15DD0141	0	0	0	0	1	1	1	0	1	1	0	0	0
15DD0160	1	0	0	0	2	0	0	0	1	1	1	1	0
16DL0615	1	0	1	0	2	0	1	0	2	1	1	1	0
14DD0147	1	0	0	0	0	0	1	0	1	1	0	1	0
16DD0186	1	0	1	0	2	0	1	0	0	1	0	1	0
15DL0124	1	0	1	0	1	0	1	0	1	1	0	2	0
13DD0918	2	0	1	0	3	0	1	0	2	2	1	1	0

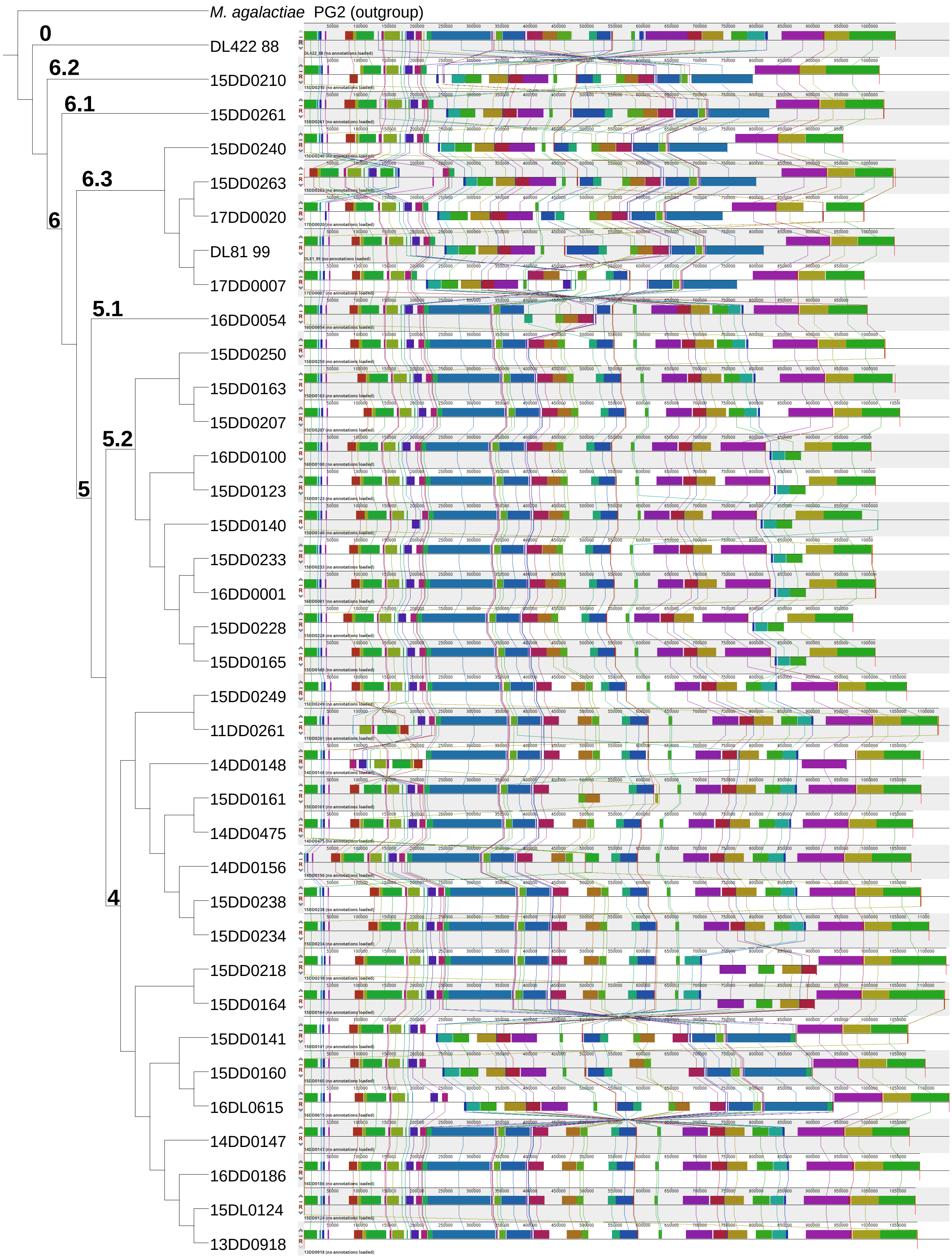

Figure S2. Recombinations within our 36 assemblies. Constructing whole-genome alignments with `Mauve` revealed the same organization in the 5' and 3' regions, but several changes in between, consistent with phylogeny. We observed rearrangements conserved in subtrees, indicating a true rearrangement and not an assembly error. The alignment of representative assemblies is shown in Fig. 4.

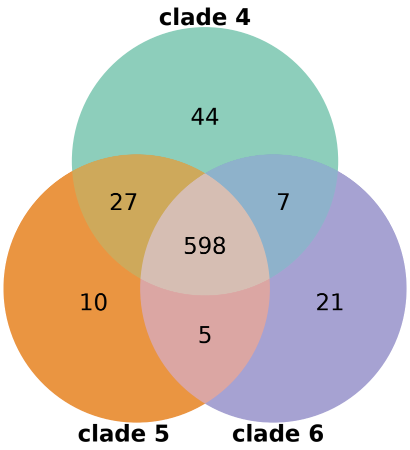

Figure S3. Schematic visualization of the pairwise gene association analysis. Pairwise gene association analysis detected 114 significant genes in total; 49 genes linked to clade 4 (44 positive and 5 negative), 17 to clade 5 (10 positive and 7 negative), and 48 to clade 6 (21 positive and 27 negative).

Table S3. Comparison of assembler based on *M. bovis* strain 17DD0007. #C – number of contigs after assembly; Total L – length of total assembly in bp; L Contg – length of longest contig in bp; GC – GC content (%)

Assembler	#C	Total L	L Contg	N50	GC
`Mycovista` with `Flye`	3	997,130	973,627	973,627	29.2
`Canu`	3	1,013,25	617,251	617,251	29.4
`Unicycler`	29	984,75	621,801	621,801	29.27

Table S4. Comparison of genome annotation tools based on *M. bovis* strain 17DD0007. CDS – number of annotated CDS (including unknown proteins); tR – number of tRNAs; rR – number of rRNAs; tm – number of tmRNAs;

Annotation	CDS	tRNA	rRNA	tmRNA
Prokka	837	34	4	1
Bakta	1902	34	4	1
